# TLR2-Deficiency Promotes Prenatal LPS Exposure-Induced Offspring Hyperlipidemia

**DOI:** 10.3389/fphys.2019.01102

**Published:** 2019-08-22

**Authors:** Dayan Cao, Wenjia Wang, Shuhui Li, Wenjing Lai, Xiaoyong Huang, Jianzhi Zhou, Xin Chen, Xiaohui Li

**Affiliations:** ^1^Institute of Materia Medica, Department of Pharmaceutics, College of Pharmacy, Army Medical University, Chongqing, China; ^2^Department of Clinical Biochemistry, College of Pharmacy, Army Medical University, Chongqing, China; ^3^Department of Pharmacy, Xinqiao Hospital, Army Medical University, Chongqing, China; ^4^Institute of Immunology, PLA, Army Medical University, Chongqing, China

**Keywords:** hyperlipidemia, prenatal lipopolysaccharide stimulation, TLR2, TLR4, VLDLR

## Abstract

Toll-like receptor 2 (TLR2), which recognizes several lipopeptides and transduces inflammatory signaling, promotes the pathogenesis of diet-induced dyslipidemia and obesity. TLR2-deficient mice were shown to have improved insulin sensitivity and reduced diet-induced metabolic syndrome. Previous studies demonstrated that prenatal lipopolysaccharide (LPS) exposure causes dyslipidemia accompanied by increased body weight and insulin resistance in offspring. To determine whether TLRs are involved in this complex abnormal phenotype, we analyzed TLR2 and TLR4 expression levels in adipose tissues from offspring with prenatal LPS-exposure (offspring-pLPS) and compared these levels to those of control offspring with prenatal saline-exposure (offspring-pSaline). TLR2 expression was specifically upregulated in the adipose tissue of offspring-pLPS mice. However, unexpectedly, TLR2-deficient offspring-pLPS mice not only presented with an abnormal phenotype comparable to that of wild-type offspring-pLPS mice but also exhibited significantly more severe hyperlipidemia. Our further analyses revealed a dramatic upregulation of TLR4 expression and overactivation of the TLR4/Myd88 signaling pathway in TLR2-deficient offspring-pLPS adipose tissue. Our finding suggests a compensatory genetic interaction between TLR2 and TLR4 in the context of prenatal inflammatory stimulation, and this interaction likely contributes to the prenatal inflammation-induced hyperlipidemia and lipid overload-induced obesity, thus providing a potential mechanism for the fetal origin of adult metabolic diseases.

## Introduction

Obesity is associated with an increased risk of coronary heart disease and diabetes, partly due to its strong association with hyperlipidemia. According to the World Health Organization (WHO) Obesity and Overweight report in 2017, more than 1.9 billion adults were overweight, with a body mass index (BMI) over 25, and in 2016, over 650 million were obese, with a BMI > 30. Despite the development of a series of measures to treat and control obesity and its related complications, the number of people who were obese or overweight increased based on the WHO report, suggesting a multifactorial contribution to the pathogenesis of obesity.

Increasing evidence and epidemiological studies support the notion that adverse intrauterine exposure to inflammation is associated with chronic diseases in adult offspring (Gluckman and Hanson, 2004; McEvoy et al., 2014; Palinski, 2014; Postma et al., 2015), including obesity (Kubo et al., 2014; Fleisch et al., 2015). We and others previously reported that prenatal LPS/zymosan exposure results in obesity in rat and mouse offspring (Nilsson et al., 2001; Wei et al., 2007; Gao et al., 2014; Qin et al., 2017). Rat offspring as young as 2 weeks old (Gao et al., 2014) and mouse offspring as young as 4 weeks old (Qin et al., 2017) with prenatal LPS exposure presented with significantly higher body weights and dyslipidemia. The obesity and hyperlipidemia in these offspring was most likely due to the enhanced differentiation and enlargement of adipocytes (Qin et al., 2017). Pyrrolidine dithiocarbamate (PDTC, a selective NF-κB inhibitor) intervention during middle gestation could successfully remediate obesity, hyperlipidemia and hypertension induced by prenatal LPS exposure (Hao et al., 2010), which was consistent with the notion in epidemiological studies that prenatal inflammatory exposure is likely associated with chronic diseases in offspring (Palinski, 2014).

In the past 2 decades, mammalian TLRs have been shown to contribute to various diseases (Medzhitov et al., 1997). Composed of an ectodomain (for ligand binding), a single transmembrane domain (for determining the receptor location), and a cytoplasmic Toll/IL-1 receptor domain (for recruitment of signaling adaptor molecules), 10 and 12 functional TLRs have been identified in humans and mice, respectively. As the major component of pattern-recognition receptors, TLRs can recognize lipoproteins, LPS, flagellin, and virus nucleic acids. Upon activation, TLRs recruit the adaptor Myd88 (myeloid differentiation factor 88) or TRIF (TIR domain-containing adapter inducing IFN-beta) to initiate downstream signal pathways (Goulopoulou et al., 2016).

It is well recognized that inflammatory pathways are activated in tissues of obese animals and humans and play an important role in obesity-associated insulin resistance. Recently, Shi et al. (2006) have shown that TLR4 is involved in the development of lipid infusion-induced insulin resistance. Additional studies have suggested that free fatty acids (FFAs) can induce TLR4-dependent insulin resistance, which requires fetuin-A as an endogenous ligand to mediate the interaction between FFAs and TLR4 (Pal et al., 2012). Interestingly, several studies have demonstrated that TLR2-deficiency attenuates local inflammatory cytokines in the liver and adipose tissue, thus protecting mice from diet-induced insulin resistance, obesity, and hepatic steatosis (Himes and Smith, 2010; Kellermayer et al., 2011), suggesting TLRs may have a general function in mediating metabolic diseases. However, whether TLRs play a role in insulin resistance and obesity in offspring with prenatal LPS exposure remains unexplored.

In the present study, we sought specifically to determine the role of TLRs in insulin resistance and obesity in offspring with prenatal LPS exposure. Contradictory to our original hypothesis, we found that TLR2-deficiency promotes, rather than prevents, dyslipidemia in prenatal LPS-induced obese offspring. Further mechanistic studies suggested that this elevated level of lipid metabolic dysfunction is associated with mal-adaptation of the overactivation of the TLR4-MYD88 pathway in prenatal LPS-induced hyperlipidemia. This work highlights unique genetic interactions among TLR-family members in promoting metabolic diseases in prenatal inflammation-induced offspring.

## Materials and Methods

### Animals

The animal surgical procedures performed in this project were approved by the Ethical Committee for Animal Experimentation of Army Medical University. TLR2-deficient mice (C57BL/6 background) were originally imported from Jackson Laboratory (stock number: 004650) and maintained with C57BL/6 for heterozygous breeders. All mice were housed in the Experimental Animal Center of the Army Medical University. All mice were maintained in specific-pathogen-free (SPF) conditions and given free access to a standard laboratory mouse chow diet and tap water. All animal experiments were conducted under the guidance of the Guide for the Care and Use of Laboratory Animals published by the US National Institutes of Health (NIH publication 86–23 revised 1985).

Prenatal LPS exposure was performed as previously described (Qin et al., 2017). In brief, virgin heterozygous female (8- to 12-week-old) mice were time-mated with heterozygous males, and the day on which a vaginal plug was detected in the mated female was defined as 0.5 days post coitum (dpc). At 11.5 dpc, female mice with a weight gain of 2 grams were considered pregnant and were subsequently intraperitoneally injected with either saline (100 μl) or LPS (75 μg/kg, Sigma, stock number: L8274). The pups were raised with the same lactating mother without LPS exposure to weaning age (4 weeks of age), at which time they were transferred to cages containing three or four pups. Only wild-type and homozygous mutant male mice were used in the study. A schematic diagram of experimental design is presented in Figure 2A.

### Diet Intervention

HFD feeding experiments were performed as previously described (Lin et al., 2000). Briefly, male offspring of C57BL/6 WT mice and congenic TLR2-deficient littermates received normal chow diet (NCD) or HFD (Research Diets Inc., stock number: D12451) starting at the age of 4 weeks. Based on genotypes, the treatment of the mother, and the types of chow consumed, the offspring were divided into eight groups: pSaline, pLPS, TLR2-deficient + pSaline, TLR2-deficient + pLPS, pSaline + HFD, pLPS + HFD, TLR2-deficient + pSaline + HFD, TLR2-deficient + pLPS + HFD. At the age of 12 weeks, the mice were sacrificed, and blood samples were collected. The serum samples were analyzed in the Department of Clinical Laboratory, Xinqiao Hospital, Chongqing, China. The epididymal white adipose tissue was dissected, weighed, and immediately frozen in liquid nitrogen or fixed in 4% paraformaldehyde for further biochemical or histological analyses.

### Histological Analysis

After 24 h of fixation in 4% paraformaldehyde, the adipose tissue was subjected to gradient dehydration, embedded in paraffin, and then sectioned and stained with hematoxylin and eosin (H&E). The morphometry of individual fat cells was assessed using digital image analysis as described previously (Qin et al., 2017). Briefly, microscopic images were digitized in 24-bit RGB (specimen level pixel size 1.28 × 1.28 μm^2^). Fat cells were recognized by applying a region-growing algorithm on manually indicated seed points and calculating the minimum Feret diameter.

### Relative Quantification of Adiposity by Micro-CT

Mice were anesthetized with 5% isoflurane and comfortably positioned in the animal holder of microcomputed tomography (micro-CT, PerkinElmer). Mice were scanned and fat compartments were quantified by Analyze 12.0 (AnalyzeDirect) according to the manufacturer’s instructions.

### Intraperitoneal Glucose Tolerance Test (IPGTT)

The IPGTT was performed during the 12th week of the experimental period. After 6 h of fasting, a glucose solution of 2 g/kg was intraperitoneally administered, and blood glucose was measured by using a glucose meter 0, 30, 60, and 120 min after obtaining blood from the tail vein. The area under the curve (AUC) was calculated.

### Intraperitoneal Insulin Tolerance Test (IPITT)

An intraperitoneal injection of 0.75 μ/kg insulin (Eli Lilly) was administered after 6 h of fasting, and blood glucose levels were measured at 0, 15, 30, and 60 min using a glucose meter. The AUC was calculated for fasting blood glucose.

### Cytokine Measurement

The serum concentrations of IL-6 and TNF-α were determined using commercial ELISA kits according to the supplier’s instructions (R&D).

### Western Blotting

The total protein of the adipose tissue was extracted and quantified using a bicinchoninic acid method. Equally loaded protein extracts were separated by sodium dodecyl sulfate-polyacrylamide gel electrophoresis. The proteins were then transferred onto PVDF membranes (0.22 μm pore size; Bio-Rad) for blocking and incubated with the primary antibodies and the corresponding secondary antibodies. The corresponding signals were detected with a chemiluminescence detection system.

### Real Time PCR

Total RNA of the adipose tissue was extracted with TRIzol according to the manufacturer’s instructions. One microgram of total RNA was reverse-transcribed using a cDNA synthesis kit (DBI Bioscience). The expression of mRNA for the indicated genes was quantified by quantitative RT-PCR with the SYBR Premix ExTaq kit (DBI Bioscience) and normalized to the expression of β-actin. The relative mRNA expression levels were calculated and compared by the 2^–ΔΔ^ ^Ct^ method. Primers used for each gene are listed in Supplementary Table S1.

### Statistical Analysis

A two-tailed, unpaired Student’s *t*-test with a 95% confidence interval was performed to analyze differences in the means of two independent groups. For multiple comparisons, two-way ANOVA (using LSD for intergroup comparison) was performed by SPSS 13.0 software. Data are shown as the mean ± SEM *P*-values less than 0.05 were considered statistically significant.

## Results

### TLR2-Deficiency Does Not Prevent Obesity and Insulin Resistance in Offspring-pLPS

**FIGURE 1:**
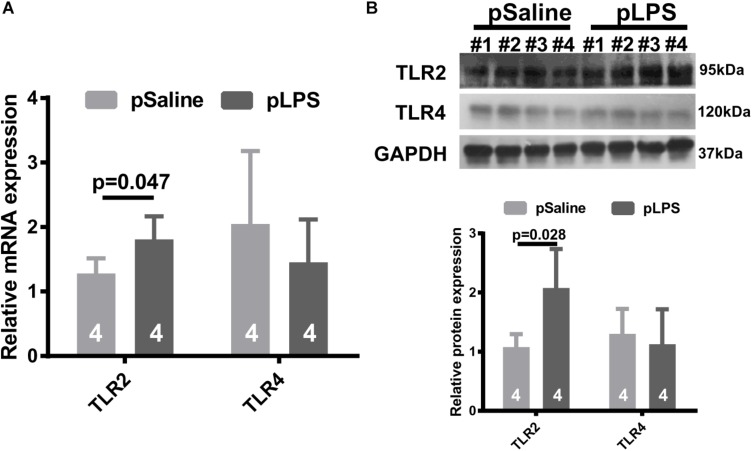
Toll-like receptor 2 (TLR2) expression is elevated in offspring with prenatal Lipopolysaccharide (LPS) exposure. Relative mRNA **(A)** and protein **(B)** expression of TLR2 and TLR4 detected by qRT-PCR and Western blot (WB) from 12-week-old offspring adipose tissue. pSaline: offspring from prenatal saline-treated mothers; pLPS: offspring from prenatal LPS-treated mothers. All data are presented as the mean ± SEM and were obtained from three independent experiments.

To test the hypothesis that TLRs contribute to the development of obesity in offspring with prenatal LPS exposure (offspring-pLPS), we analyzed the mRNA expression levels of two well-studied TLRs, TLR2, and TLR4. As shown in Figure 1A, TLR2 mRNA level was significantly elevated in the adipose tissue isolated from offspring-pLPS mice compared to the offspring-pSaline controls, while the mRNA levels of TLR4 remained unchanged between the two groups. Western blot analysis further confirmed this finding that TLR2 was significantly elevated in the adipose tissue of offspring-pLPS (Figure 1B). Because LPS cannot cross the placental barrier, we believe that the altered expression of TLR2 in the offspring-pLPS mice was not caused by the direct binding of LPS to TLR2 in prenatal stages, but, instead, was a secondary response in the offspring.

**FIGURE 2:**
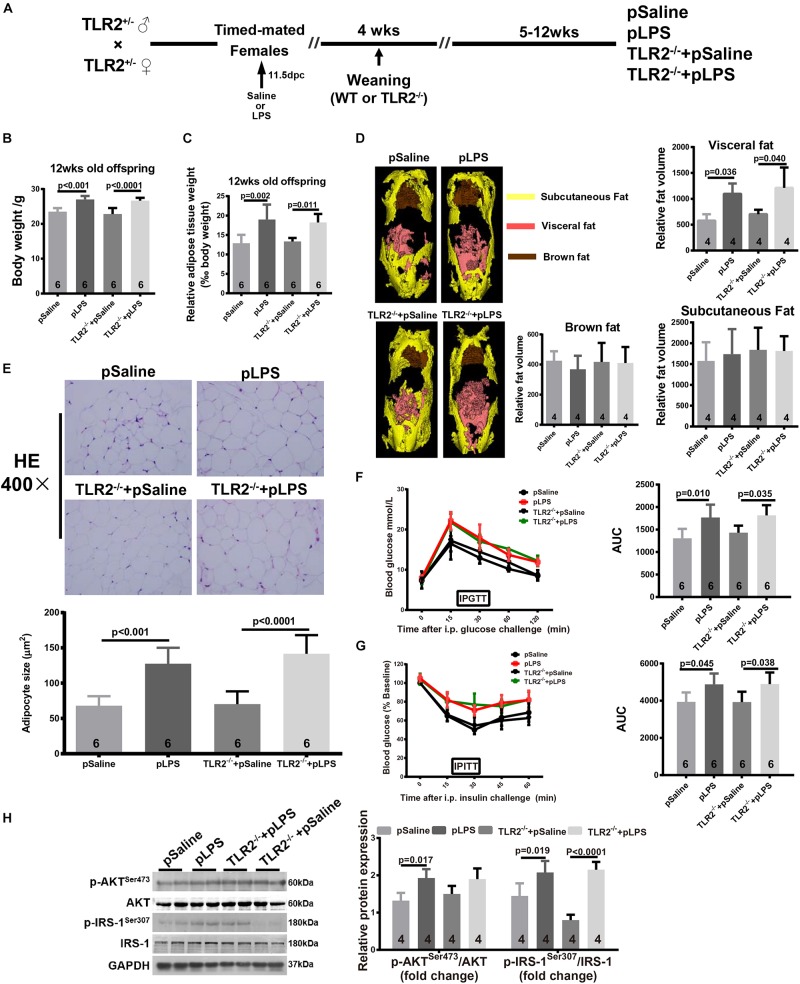
TLR2 deficiency does not prevent prenatal LPS exposure-induced obesity and insulin resistance. **(A)** Schematic diagram of experimental design. **(B)** Bodyweight of 12-week-old offspring of the indicated group. **(C)** The 12-week-old offspring relative adipose tissue weight was calculated as: (wet adipose tissue weight/body weight) × 1000. **(D)** Fat distribution was detected by micro-CT and data were processed with Analyze 12.0. **(E)** Representative adipose tissue H&E staining. Adipocyte size was calculated as described in the Methods section. **(F,G)** Intraperitoneal Glucose Tolerance Test (IPGTT) and Intraperitoneal Insulin Tolerance Test (IPITT) were performed on indicated mice. **(H)** Protein expression of AKT, IRS-1, p-AKT^ser473^, and p-IRS-1^Ser307^ were detected by WB. All data are presented as the mean ± SEM and were obtained from three independent experiments. pSaline: offspring from prenatal saline-treated mother; pLPS: offspring from prenatal LPS-treated mother; TLR2^–/–^ + pLPS: offspring from prenatal LPS treatment homozygous mated TLR2^–/–^ mother; TLR2^–/–^ + pSaline: offspring from prenatal Saline treatment homozygous mated TLR2^–/–^ mother.

To determine whether the elevated TLR2 expression was the cause of obesity and insulin resistance in the offspring-pLPS mice, we further tested whether TLR2-deficiency reversed the obesity and insulin resistance in the offspring-pLPS mice (Figure 2A). We measured the body weight (Figure 2B), relative epididymal fat weight and volume (Figures 2C,D), and adipose cell size (Figure 2E) in the offspring-pLPS and control animals. As shown in Figure 2, the offspring of both wild-type and TLR2-deficient mice prenatally treated with LPS developed similar obese phenotypes compared to control offspring prenatally treated with saline (offspring-pSaline) of either wild-type or TLR2-deficient mice at 12-weeks of age. We also found a similar trend in glucose and insulin resistance, as indicated by IPGTT, IPITT and protein expression of phosphorylated IRS-1^Ser307^ and AKT^Ser473^, in offspring-pLPS mice of both wild-type and TLR2-deficient genotypes when compared to the offspring-pSaline mice of both wild-type and TLR2-deficient genotypes (Figures 2F–H).

### TLR2-Deficiency Does Not Prevent High-Fat Induced Obesity and Insulin Resistance in Offspring-pLPS

**FIGURE 3:**
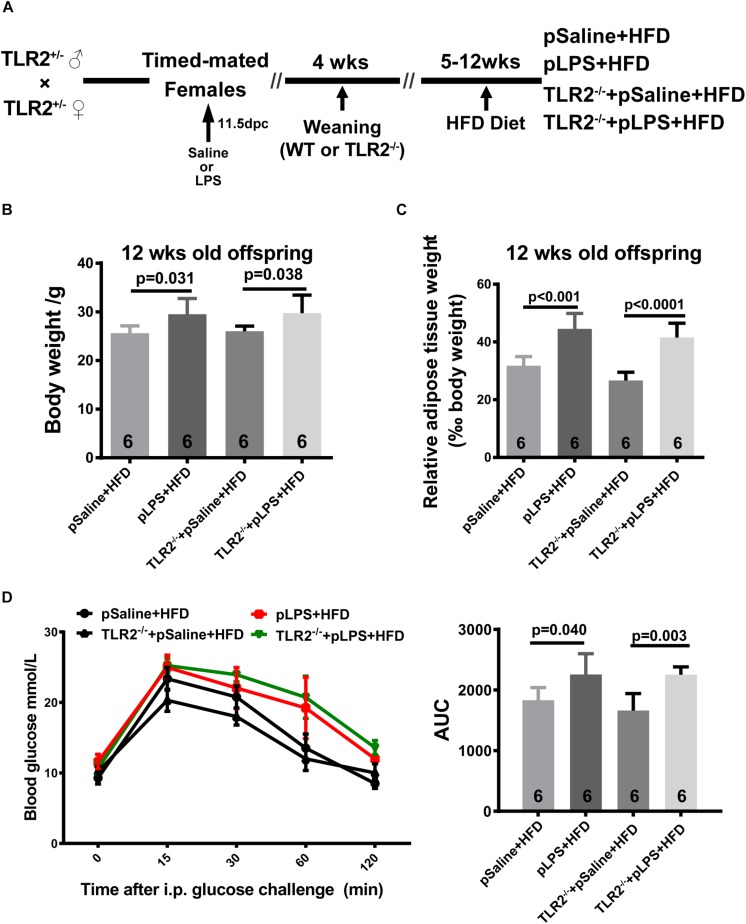
TLR2 deficiency does not prevent high-fat induced obesity and insulin resistance in offspring with prenatal LPS exposure fed a high-fat diet. **(A)** Schematic diagram of experimental design on diet intervention. **(B)** Bodyweight of 12-week-old offspring after diet intervention. **(C)** The 12-week-old diet intervened offspring relative adipose tissue weight was calculated as wet adipose tissue weight in % body weight. **(D)** IPGTT was performed, and the area under the curve (AUC) for glucose was calculated for 12-week-old diet intervened offspring. All data are presented as the mean ± SEM.

As shown in previous studies, TLR2-deficiency in adult mice improves insulin sensitivity, attenuates adipocyte hypertrophy, and reduces inflammatory cytokine expression in mice that were fed HFD (Himes and Smith, 2010; Kellermayer et al., 2011); as such, we hypothesize that postnatal HFD-induced obesity and insulin resistance is reduced in TLR2-deficient offspring-pLPS. Thus, we compared HFD-treated wild-type offspring-pLPS and HFD-treated TLR2-deficient offspring-pLPS animals. We began HFD feeding at 4 weeks of age for a duration of 8 weeks (Figure 3A). As expected, the wild-type offspring-pLPS mice fed a HFD had significantly higher body weights than HFD-fed offspring-pSaline control mice (Figure 3B). Surprisingly, TLR2-deficiency did not alter the phenotype, as the HFD-fed TLR2-deficient offspring-pLPS mice had a similarly increased level of body weight compared to HFD-fed wildtype offspring-pLPS mice (Figure 3B). Consistent with this finding, all HFD-fed offspring-pLPS of wild-type and TLR2-deficient mice presented with a similarly elevated level of relative adipose tissue weight and impaired glucose tolerance curve compared to HFD-fed pSaline controls of either genotype (Figures 3C,D). This result suggests that TLR2-deficiency did not prevents obesity and insulin resistance in prenatally treated LPS animals fed a HFD.

### TLR2-Deficiency Promotes Hyperlipidemia in Offspring-pLPS

**FIGURE 4:**
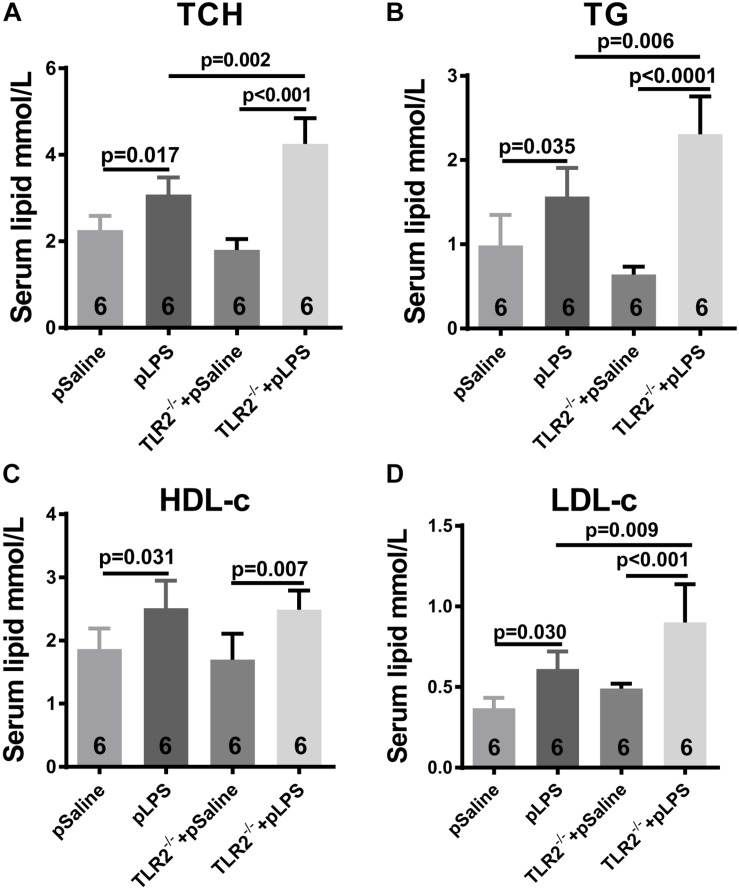
TLR2 deficiency promotes hyperlipidemia induced by prenatal LPS exposure. Measurement of serum content of TCH **(A)**, TG **(B)**, HDL-c **(C)** and LDL-c **(D)** in 12-week-old offspring. All data are presented as the mean ± SEM.

Hyperlipidemia is commonly associated with obesity and insulin resistance. Hyperlipidemia is one of the most common forms of dyslipidemia and is involved in abnormal upregulation of the levels of any or all lipids or lipoproteins in the circulatory system. To determine whether the upregulated level of TLR2 contributes to the elevated level of lipids seen in offspring-pLPS, we analyzed serum lipid content of TLR2-deficient offspring-pLPS. As shown in Figures 4A–C, unexpectedly, our data demonstrated more severely elevated serum levels of TCH, TG, and LDL-c in TLR2-deficient offspring-pLPS compared to saline and LPS treated wild-type control mice. TLR2-deficiency alone had no effect on blood lipid levels (Figure 4). The elevation of HDL-c appeared only relevant to prenatal LPS exposure (Figure 4D), suggesting a protective feedback in response to dyslipidemia induced by prenatal inflammatory exposure.

### Compensatory TLR4 Activation in TLR2-Deficient Offspring With Prenatal LPS Exposure

**FIGURE 5:**
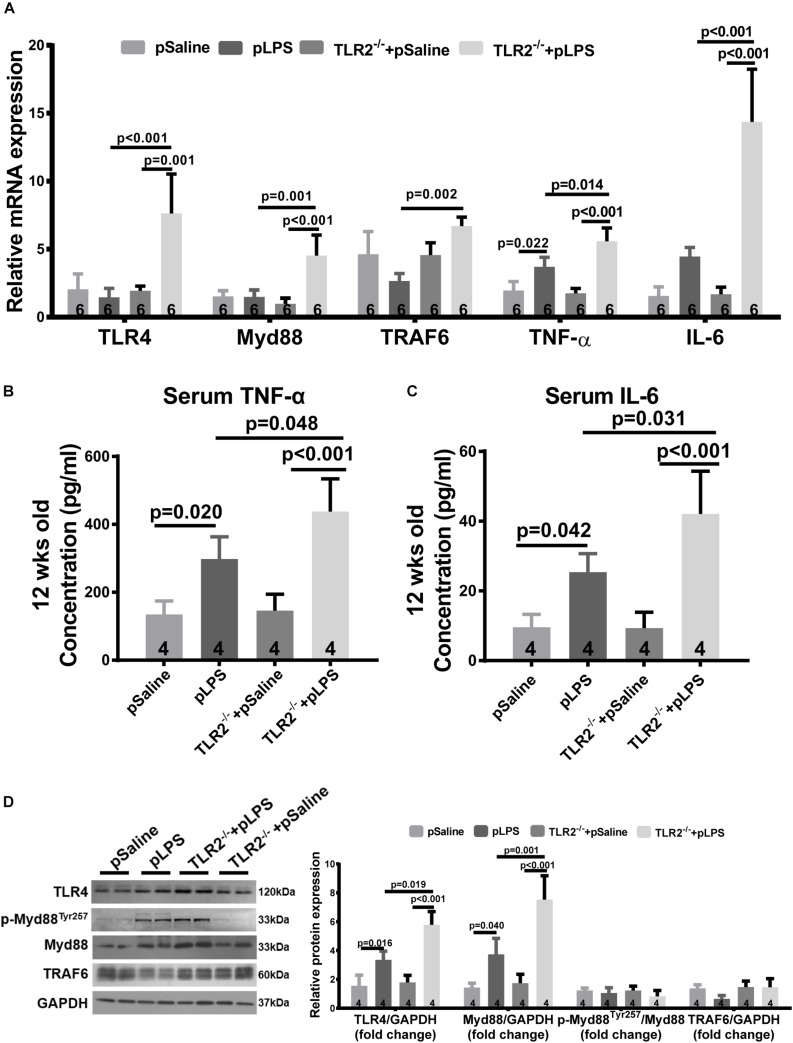
Compensatory TLR4 activation in TLR2-knockout offspring with prenatal LPS exposure. **(A)** qRT-PCR analyzing relative mRNA expression of TLR4, Myd88 TRAF6, TNF-α, and IL-6 in adipose tissue of 12-week-old offspring under different treatments. Serum TNF-α **(B)** and IL-6 **(C)** concentration were determined. **(D)** WB analyzing relative protein expression of TLR4, Myd88, p-Myd88^Tyr257^, and TRAF6 in adipose tissue of 12-week-old offspring. All data are presented as the mean ± SEM.

As TLR4 is known to contributed to hyperlipidemia (Kuwabara et al., 2012; Pal et al., 2012), we wondered whether the more severe dyslipidemia phenotype in TLR2-deficient offspring-pLPS was due to TLR4 activity. Thus, we analyzed TLR4 expression in TLR2-deficient mice using qRT-PCR. Our data showed that TLR4 expression was dramatically increased in TLR2-deficient offspring-pLPS when compared to wild-type offspring-pLPS and TLR2-deficient offspring-pSaline mice (Figure 5A). This suggests that, in the context of prenatal inflammatory stimulation, TLR4 is overactivated to compensate for the loss of TLR2, which likely contributes to the more severe hyperlipidemia phenotype in TLR2-deficient offspring-pLPS.

Myeloid differentiation factor 88 is known to be a key adaptor for TLR4-mediated signaling and is transcriptionally upregulated by numerous pro-inflammatory signal transduction pathways (Goulopoulou et al., 2016). To determine whether TLR4-mediated signaling was indeed overactivated, we analyzed Myd88 expression level in TLR2-deficient offspring-pLPS adipose tissue. Consistent with the upregulation of TLR4, the Myd88 transcript was highly elevated in TLR2-deficient offspring-pLPS (Figure 5A). Additionally, the serum concentration of cytokines for TLR4 activation, such as TNF-α and IL-6, were also significantly elevated in TLR2-deficient offspring-pLPS (Figures 5B,C). Western blot analysis further confirmed a significant elevation of TLR4 and Myd88 protein levels in TLR2-deficient offspring-pLPS, while the expression of phospho-MyD88^Tyr257^ was proportionally increased when normalized to total Myd88 protein levels in the TLR2-deficient offspring-pLPS adipose tissue (Figure 5D), suggesting that the activation of MyD88 is at the transcriptional level. Since the phosphorylation of Myd88 at Tyr257 is likely involved in the recruitment of PI-3 kinase p85 subunit to Myd88 for further inflammatory signal transduction, our results suggest that this process might not be affected by the genetic compensation of TLR4 alone, however, our results highlight the potential hypersensitivity in TLR2-deficient offspring-pLPS to additional inflammatory stimulation (Gelman et al., 2006; Heun et al., 2019).

### Increased High-Mobility Group Box 1 (HMGB-1) Expression in TLR2-Deficient Offspring-pLPS

**FIGURE 6:**
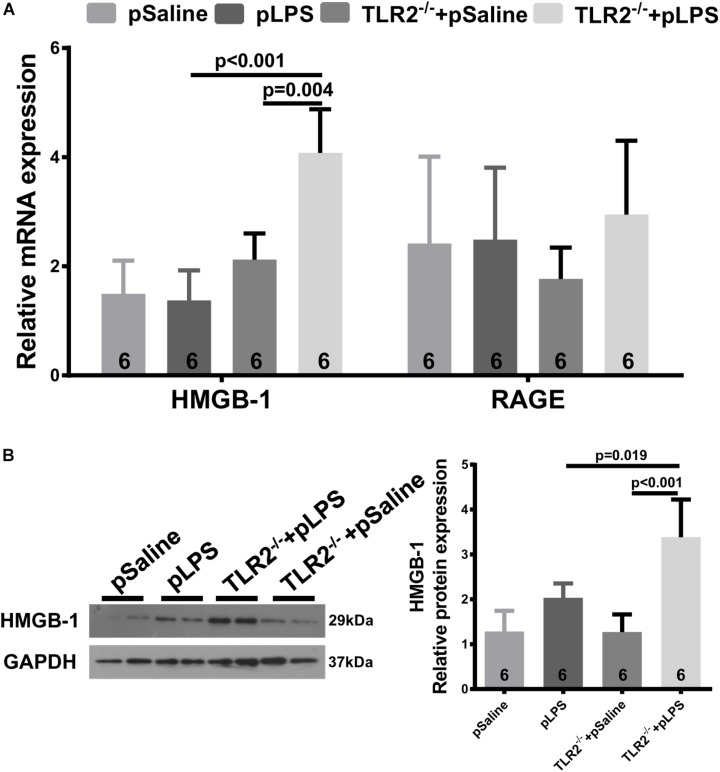
Upregulated HMGB-1 expression in TLR2-deficient offspring with prenatal LPS exposure. **(A)** qRT-PCR analyzing relative mRNA expression of HMGB-1 and RAGE in adipose tissue of 12-week-old offspring. **(B)** Western blot analyzing relative protein expression of HMGB-1 in adipose tissue of 12-week-old offspring. All data are presented as the mean ± SEM.

High-Mobility Group Box 1, a well-known damage associated molecular pattern (DAMP), is a highly conserved non-histone nuclear protein that is known to play pivotal roles in the pathogenesis of metabolic diseases and related chronic inflammation (Wu et al., 2016; Zhang et al., 2017). HMGB-1 can be upregulated by necrotic cells during tissue injury or by immune cells upon inflammatory stimulation, and it acts as an inflammatory cytokine (Andersson et al., 2018). It was reported that hyperlipidemia upregulates the expression of HMGB-1 in experimentally induced hyperlipidemia (Haraba et al., 2011). Thus, we analyzed HMGB-1 expression in the TLR2-deficient offspring-pLPS adipose tissue. As shown in Figure 6, both qRT-PCR and Western blot analyses demonstrated that HMGB-1 expression levels were significantly elevated in the TLR2-deficient offspring-pLPS adipose tissue. Extracellular HMGB-1 is known to interact with multiple receptors (Chan et al., 2012), including the receptor for advanced glycation end products (RAGE) and TLR4. RAGE expression was not affected in these mice (Figure 6A), suggesting that HMGB/TLR4 is the major signaling component of inflammation in TLR2-deficient offspring-pLPS.

### VLDLR Expression in TLR2-Deficient Offspring-pLPS

**FIGURE 7:**
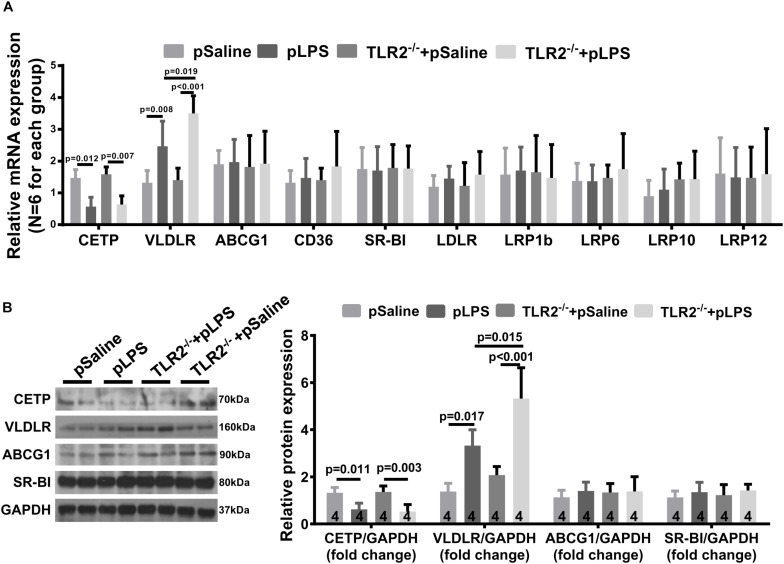
Very Low Density Lipoprotein Receptor (VLDLR) expression in TLR2-deficient mice with prenatal LPS exposure. **(A)** qRT-PCR analyzing relative expression of Cholesteryl Ester Transfer Protein (CETP), VLDLR, ABCG1,CD36, SR-BI, LDLR, LRP1b, LRP6, LRP10, and LRP12 in adipose tissue of 12-week-old offspring. **(B)** Western blot analyzing relative expression of CETP, VLDLR, ABCG1, and SR-BI in adipose tissue of 12-week-old offspring. All data are presented as the mean ± SEM.

One puzzling dilemma is that TLR2-deficiency significantly promotes hyperlipidemia in offspring-pLPS without enhancing the severity of obesity and insulin resistance in offspring-pLPS. We assumed that this was due to the short experimental duration. However, this phenomenon also suggests that there could be a protective feedback in response to the hyperlipidemia which prevents worsening of the abnormal phenotype. To test this hypothesis, we analyzed gene expression of a panel of lipoproteins considered to be involved in the pathogenesis of hyperlipidemia; this panel included lipoproteins such as reverse cholesterol transporters [e.g., ATP-binding cassette subfamily G member 1 (ABCG1), cholesteryl ester transfer protein (CETP), CD36 and scavenger receptor class B-member 1 (SR-BI)] and the low density lipoprotein receptor (LDLR) family members [e.g., LDLR, low density lipoprotein-related protein 1B (LRP1b), low density lipoprotein receptor-related protein 6 (LRP6), low-density lipoprotein receptor-related protein 10 (LRP10), low density lipoprotein-related protein 12 (LRP12) and very low density lipoprotein receptor (VLDLR)] (Stewart et al., 2010). We found significantly downregulated CETP expression in offspring-pLPS independent of the TLR2 genotype, which might partly contribute to the dyslipidemia phenotype (Figures 7A,B). More importantly, we observed a specific upregulation of VLDLR at both the mRNA and protein levels in offspring-pLPS, and its expression was further elevated in TLR2-deficient offspring-pLPS adipose tissue (Figures 7A,B). As VLDLR is known to reduce the blood cholesterol level (Turunen et al., 2016), this upregulation of VLDLR is likely a protective feedback response due to hyperlipidemia induced by prenatal inflammatory stimulation, which might explain the relatively mild obesity and insulin resistance phenotype in offspring-pLPS and TLR2-deficient offspring-pLPS.

## Discussion

Growing evidence has shown that intrauterine exposure to adverse conditions can lead to predisposition for chronic diseases (Gluckman and Hanson, 2004; McEvoy et al., 2014; Palinski, 2014; Postma et al., 2015). In the present study, we successfully created an animal model in which offspring obesity can be induced by prenatal LPS exposure. TLR2 expression is elevated in offspring with prenatal LPS exposure. As TLR2-deficiency was previously shown to prevent the pathogenesis of obesity, we initially hypothesized that the TLR2-deficiency would rescue prenatal LPS exposure-induced offspring obesity. However, surprisingly, our observations demonstrated the opposite; TLR2-deficiency did not prevent prenatal LPS stimulation-induced obesity, and it instead promoted hyperlipidemia. Our study revealed a genetic compensatory interplay between TLR2 and TLR4 in the context of prenatal inflammatory stimulation.

It has been shown that TLR2 and TLR4 share common ligands and similar functions in various physiological and pathological conditions (Jiang et al., 2005; Erridge, 2010; Hwang et al., 2016). For example, dietary saturated fatty acid [e.g., lauric acid (Lee et al., 2001, 2004) and palmitic acid (Shi et al., 2006; Snodgrass et al., 2013)] are able to activate either TLR2 and/or TLR4 and lead to NF-κB activation. One important finding from the present study is that TLR4-Myd88 overactivation leads to more severe adipose metabolic dysfunction, suggesting that TLR4 plays a more dominant role in lipid metabolism in response to inflammation. This finding is consistent with a previous study in which pharmacological inhibition of TLR4 was found to significantly lower the serum concentration of cholesterol and triglycerides in normal mice (Lu et al., 2013). Moreover, several epidemiological investigations revealed that HMGB-1 (one of the TLR4 ligands) can serve as an important diagnostic marker for insulin resistance and obesity (Arrigo et al., 2013; Gunasekaran et al., 2013; Subramanian et al., 2017). The use of anti-HMGB1 neutralizing antibody effectively reduce HFD-induced weight gain and liver inflammation in mice (Montes et al., 2015), suggesting that TLR4 is likely a key therapeutic target for dyslipidemia and obesity. Our study nevertheless supports these previous observations.

The LDLR family members are known to be important in regulating the pathogenesis of hyperlipidemia (Go and Mani, 2012). Among all of the detected genes in the LDLR family, we found that VLDLR expression in adipose tissue is significantly upregulated in offspring-pLPS and is further elevated in TLR2-deficient offspring-pLPS (Figures 6A,B). Considering that VLDLR is important in postprandial chylomicron and VLDL clearance (Goudriaan et al., 2004), the upregulation of VLDLR is thought to lower serum lipids, which is opposite of the hyperlipidemia phenotype seen in both offspring-pLPS and TLR2-deficient offspring-pLPS. This phenomenon may explain the relatively mild obesity and insulin resistance phenotype in both offspring-pLPS and TLR2-deficient offspring-pLPS. The upregulation of VLDLR can be interpreted as protective feedback due to the higher lipid content induced by prenatal inflammatory stimulation or, alternatively, in direct response to prenatal inflammatory stimulation. Previous studies have revealed that VLDLR expression is regulated by peroxisome proliferator-activated receptor-gamma (PPAR-γ) (Takazawa et al., 2009; Tao et al., 2010). PPAR-γ is a key transcriptional factor in both inflammation response and lipid metabolism (Glass and Saijo, 2010; Lodhi and Semenkovich, 2014; Kang et al., 2016). Our future studies will determine whether PPAR-γ is a key regulator involved in the regulation of VLDLR expression in our prenatal inflammatory stimulation models.

Although it was not determined in the present study, we must mention the potential effect of gut microbiota alteration caused by prenatal LPS stimulation. The infant microbiome, which plays an essential role in adult health and diseases, is determined by maternal-offspring exchanges of microbiota (Mueller et al., 2015). A recent study found that maternal poly (I:C) exposure results in behavioral abnormalities that require the participation of defined gut commensal bacteria with a propensity to induce TH17 cells (Kim et al., 2017).

Finally, our study provides an important model system for the fetal origin of adult disease (FOAD), specifically in disorders of lipid metabolism and obesity. We believe further exploration of such a fetal inflammatory response will provide novel insights into obesity and other chronic diseases, such as atherosclerosis and related cardiovascular diseases.

## Conclusion

In conclusion, the present study suggests that a compensatory genetic interaction between TLR2 and TLR4 contributes to the prenatal LPS stimulation-induced hyperlipidemia and lipid overload-induced obesity. Our study describes a unique molecular network between the genotype and phenotype relationship in lipid metabolism and a different angle to understand potential metabolic and other related diseases.

## Data Availability

The raw data supporting the conclusions of this manuscript will be made available by the authors, without undue reservation, to any qualified researcher.

## Ethics Statement

All animal experiments were conducted under the guidance of the Guide for the Care and Use of Laboratory Animals published by the United States National Institutes of Health (NIH publication 86–23 revised 1985). The animal surgical procedures performed in this project were approved by the Ethical Committee for Animal Experimentation of Army Medical University.

## Author Contributions

DC, JZ, SL, and XL designed the experiments and drafted and revised the manuscript. DC, WW, XC, WL, and XH carried out the experiments, analyzed the data and revised the manuscript. All authors reviewed the manuscript and approved the manuscript that was submitted for publication.

## Conflict of Interest Statement

The authors declare that the research was conducted in the absence of any commercial or financial relationships that could be construed as a potential conflict of interest.
