# Supplementary material for: TLR2-Deficiency Promotes Prenatal LPS Exposure-Induced Offspring Hyperlipidemia
Source: Front Physiol. 2019 Aug 22;10:1102. doi: 10.3389/fphys.2019.01102 (PMC6713936; doi:10.3389/fphys.2019.01102)
Supplement: Supplementary file 1 [file Table_1.DOCX]

Supplementary Material

Supplementary Information Table 1. The sequences of PCR *primers used* *in* *this* *manuscript* are displayed as following:

| **Gene** | **Sense** | **Anti-sense** |
| --- | --- | --- |
| TLR2 | AAGGAGGTGCGGACTGTTTC | GAGCCAAAGAGCTCGTAGC |
| TLR4 | AGTGAATGAGCCCCAGCAAA | TGCAAGGAGGTTCAGTGCTC |
| Myd88 | CATACCCTTGGTCGCGCTTA | CCAGGCATCCAACAAACTGC |
| TRAF6 | TGAGTGTAGCCCACGAAAGC | CACCCCAGCAGCTAAGAACA |
| IL-6 | CCCCAATTTCCAATGCTCTCC | GGATGGTCTTGGTCCTTAGCC |
| TNF-α | GGCAGTTAGGCATGGGATGA | TACCTACGACGTGGGCTACA |
| HMGB-1 | GGAGAAACTTCAGACCGGACG | CATCAAACTTTGCTCGGGCG |
| RAGE | TGACTGGGTTCACAAGGAAGG | CGATCTGGGTGCTCTTACGG |
| VLDLR | GTGCCTGAGCTGCTGGG | CTTGCACACTCTACTCCCCG |
| CETP | CCTGGTGTTGAACCACGAGA | CTGGATGTTGACTTGACTTGG |
| ABCG1 | CCTGCCTCCTCTTCTACCCT | TGCCTTGGGTTTGGGTTTCT |
| SR-BI | TTCGAACAGAGCGGAGCAAT | TCAGAGTAGGCCTGAATGGC |
| LDLR | CAGGTACTGGCAACCACCAT | TTTGGAGGATGAGAACCGGC |
| CD36 | CCTAGTAGGCGTGGGTCTGA | CACGGGGTCTCAACCATTCA |
| Lrp1b | CGTGCCACTTGTCCTTTTGG | GGTGCTGTATGTGGGAGCTT |
| Lrp6 | CCGACAAGTCGAGAACCCAT | TGCTGAACGAACAGAGTAGG |
| Lrp10 | AAAGCCGCCGTCCCCTA | CAAACTTTGTGGACTGCCCG |
| Lrp12 | TGTCCAGGAAAGGCTTCAGG | GGGACAGACACTGGAACTGG |
